# Supplementary material for: Nosological and Theranostic Approach to Vascular Malformation through cfDNA NGS Liquid Biopsy
Source: J Clin Med. 2022 Jun 28;11(13):3740. doi: 10.3390/jcm11133740 (PMC9267326; doi:10.3390/jcm11133740)
Supplement: Supplementary file 1 [file jcm-11-03740-s001.zip › jcm-1757789-supplementary.pdf]

Supplementary Material: genes panel analyzed in gDNA NGS

GNA11 (OMIM #139313; Ref Seq NM_002067.4; Assembly GRCh37/hg19),

GNAQ (OMIM #60998; Ref Seq NM_002072.4; Assembly GRCh37/hg19),

AKT1 (OMIM #164730; Ref Seq NM_005163.2; Assembly GRCh37/hg19),

KRAS (OMIM #190070; Ref Seq NM_033360.3; Assembly GRCh37/hg19),

HRAS (OMIM #190020; Ref Seq NM_005343.3; Assembly GRCh37/hg19),

IDH1 (OMIM #147700; Ref Seq NM_005896.3; Assembly GRCh37/hg19),

PIK3CA (OMIM #171834; Ref Seq NM_006218.3; Assembly GRCh37/hg19),

TEK (OMIM #60221; Ref Seq NM_000459.4; Assembly GRCh37/hg19),

PTEN (OMIM #601728; Ref Seq NM_000314.7; Assembly GRCh37/hg19),

RASA1 (OMIM #139150; Ref Seq NM_002890.2; Assembly GRCh37/hg19),

TGFBR2 (OMIM #190182; Ref Seq NM_001024847.2; Assembly GRCh37/hg19),

TGFBR1 (OMIM #190181; Ref Seq NM_004612.3; Assembly GRCh37/hg19),

SMAD3 (OMIM #603109; Ref Seq NM_005902.3; Assembly GRCh37/hg19),

SMAD4 (OMIM #600993; Ref Seq NM_005359.5; Assembly GRCh37/hg19),

TGFB1 (OMIM #190180; Ref Seq NM_000660.6; Assembly GRCh37/hg19),

TGFB2 (OMIM #190220; Ref Seq NM_001135599.3; Assembly GRCh37/hg19),

GLMN (OMIM #601749; Ref Seq NM_053274.2; Assembly GRCh37/hg19).

Supplementary Table S1: Clinical features of the patients. The table reports the patient's identification number, sex, age and their clinical features.

Patient	Gender	Age	Clinical Features
1	F	64	Angiodysplasia and hypertrophy of the right lower limb. Multiple tumors.
2	F	34	Lower limb Port-Wine Stain angyoma and vascular malformation. The malformation needed an amputation. The patient also suffered from pancreatic neoplasm.
3	F	58	Fetal overgrowth. Oropharyngeal and facial angiomas. Vascular malformation with hypertrophy of the left lower limb. Uterine angioma.
4	M	38	Multiple superficial and deep vascular malformations of the left lower limb with extension to the gluteus maximus and ankle. Left knee angioma. Multiple splenic angiomas-amarthromas. Atrial septal aneurysm. Patent foramen ovale.
5	F	26	Angiomas in the right hemisome, particularly in the limbs. Hypertrophy of the right lower limb.
6	M	38	Angiodysplasia and hypertrophy of the left lower limb.
7	M	6 days	Angiomatous lesion affecting the left side prevalent in the left lower limb, with muscle infiltration. Patent Botallo Duct with left right shunt.
8	F	11 months	Scapular, upper limb, hand, and sternal capillary malformation (port-wine stain). The patient also suffers from Alport syndrome (COL4A4).
9	F	23	Right lower limb venous malformation and hypertrophy. Four angiomas on the right knee.
10	F	18	Lower limbs and lumbar vascular lesions. Right thigh angiodysplasia. Hypoplasia of the superior femoral vein.
11	M	64	Angiodysplasia of the right lower limb with elephantiasis. Intestinal lymphangiomatosis.

12	M	56	Arteriovenous fistulas in the right upper limb and in the right hemithorax and presence of ectatic vessels. Tortuosity of the right subclavian artery. The patient also presented with subarachnoid haemorrhage.
13	F	26	Angiomas on both hands, in the abdomen and in the left lower limb.
14	M	42	Venous malformation on the left side of the neck with extension to the submandibular level. Reduction of the oropharyngeal airspace. Localization also at the level of the left knee. Colon angioma
15	F	37	Right hand telangectasias. Paravertebral, chest, and forearm, complex vascular malformation of mixed-capillary-lymphatic-venous aspect. The lesion infiltrates the muscles. Extended area of osteopenia to the distal third of the diaphyseal radius and ulna
16	F	34	Upper right limb and chest angiomatosis. Upper right limb, hand, and III-IV fingers hypertrophy. Right thigh varicosity.
17	F	25	Cavernous angiomas in the right hand, right forearm, cerebellum, spleen and thorax with thrombotic complications.
18	M	33	Left upper limb and hemithorax arteriovenous malformation. The lesion expansion caused pulmonary hypoexpansion and functional impotence of the limb
19	F	42	Vascular malformation in the wrist
20	F	40	Dorsal angiomas, rectal cavernous hemangioma, frontal cortical cavernous hemangioma.
21	M	37	Posterior right lower limb and scrotum venous malformation.
22	F	49	Right upper limb and hand glomuvenous malformations.
23	M	14	Proliferation of arterial, venous and ectatic vessels in the vastus medial right muscle and presence of angiomatosis. Coiling-kinking of the internal carotid artery for a dysplastic condition. Hypertrophy of the right lower limb.
24	M	18	Microfistulas of the left lower limb. Lower limb dysmetry.

25	M	41	Multiple chest angiomatosis and telangiectasias. Vertebral and splenic angiomas.
26	F	37	Glomus venous malformation of the right upper limb. Abnormal vascular areas in the subcutis. At 11 years old aneurysmal bone cyst of the right fibula.
27	M	51	Complex arteriovenous malformation of the right upper limb and right hemithorax. Lymphangioma of the right upper limb.
28	M	35	Right lower limb and right thigh arteriovenous malformations.
29	M	45	Arteriovenous fistulas of the left side of the face.
30	M	56	Right calf angyoma and varicosity.
31	F	34	Right lower limb angiodysplasia that infiltrates the skin, the muscles and the bone. Right lower limb hypertrophy.
32	M	29	Angiodysplasia in the left lower limb, with extension up to the inguino-abdominal region
33	F	45	Arteriovenous malformation of the right upper limb.
34	F	40	Arteriovenous malformation of the left lower limb. Thrombosis at the age of 11.
35	M	47	Arteriovenous malformation of the right vertebral and femoral vessels. Right foot ulcer.
36	M	46	Arteriovenous malformation of some pelvic vessels. Vertebral instability
37	F	24	Right axillary arteriovenous malformation

38	M	7 months	Diffuse angioma of the thorax and left upper limb.
39	F	5	Ectasia of the left saphenous vein and the left superficial epigastric vein. Mild hypertrophy of the left lower limb.
40	F	26	Angioma and hypertrophy in the left lower limb which caused problems in walking.
41	F	61	Vascular lesion in the right lower limb, right foot and labia majora. Angiodysplasia of the sigmoid.
42	M	27	Angioma on the left cheek and multiple skin lesion on the forearm. The patient also suffers from intellectual disability.
43	M	40	Pectoral muscle lymphangioma and hemangioma at birth. At 38 years old skull osteolytic lesions. Biopsy of the lesions concluded for vascular malformation/haemangioma/lymphangioma.
44	M	60	Lower right limb angiodysplasia that infiltrates the muscles.
45	F	30	Venous malformation of the left lower limb extending to the chest. Dislocated kidney and spleen.
46	F	40	Multiple angiomas of the neck and gastrointestinal tract. She also suffered from left breast ductal cancer.
47	M	18	Venous malformation of the right lower limb.
48	F	17	Hypertrophy of the left lower limb and macrodactyly of the left big toe. Capsular and pericapsular lipomatosis of the left lower limb. Shunt of the popliteal lymphatic vessels.
49	F	32	Cavernous hemangioma in the right periscapular area
50	F	2	Abdominal and back freckles and asymmetry of the lower limbs at birth. At two years the patient presented a right lower limb malformation (venous dysplasia).

51	M	73	Fistula between the inferior mesenteric artery and the inferior mesenteric vein.
52	F	40	Arteriovenous malformation of the right thigh.
53	M	42	Vascular malformation of the right face.
